# Does the frontal sensory organ in adults of the hoplonemertean *Quasitetrastemma stimpsoni* originate from the larval apical organ?

**DOI:** 10.1186/s12983-019-0347-4

**Published:** 2020-01-06

**Authors:** Timur Yu Magarlamov, Vyacheslav Dyachuk, Alexey V. Chernyshev

**Affiliations:** 1 A.V. Zhirmunsky National Scientific Center of Marine Biology, Far Eastern Branch, Russian Academy of Sciences, Vladivostok, 690041 Russia; 2 Far Eastern Federal University, Vladivostok, 690090 Russia; 3 Department of Neuroscience, Karolinska Institute, Stockholm, Sweden

**Keywords:** Apical organ, CLSM, Frontal gland, Frontal organ, Larva, Nemertea, TEM

## Abstract

**Background:**

The apical organ is the most prominent neural structure in spiralian larvae. Although it has been thoroughly investigated in larvae of the class Pilidiophora in phylum Nemertea, studies on its structure in other nemertean larvae are limited. Most adult hoplonemertean worms have a frontal organ located in a position corresponding to that of the larval apical organ. The development and sensory function of the frontal organ has not been thoroughly characterized to date.

**Results:**

The apical organ in the early rudiment stage of *Quasitetrastemma stimpsoni* larvae consists of an apical plate enclosed by ducts of frontal gland cells and eight apical neurons. The apical plate is abundantly innervated by neurites of apical neurons. During the late rudiment stage, the larval apical organ has external innervation from below by two subapical-plate neurons, along with 11 apical neurons, and its plate contains serotonin-like immunoreactive (5-HT-lir) cells. In the vermicular stage (free-swimming juvenile), the number of apical neurons is reduced, and their processes are resorbed. Serotonin is detected in the apical plate with no visible connection to apical neurons. In adult worms, the frontal organ has a small apical pit with openings for the frontal gland ducts. The organ consists of 8 to 10 densely packed 5-HT-lir cells that form the roundish pit.

**Conclusions:**

Although the ultrastructure of the *Q. stimpsoni* larval apical organ closely resembles that of the apical organ of Polycladida larvae, the former differs in the presence of flask-shaped neurons typical of Spiralia. Significant differences in the structure of the apical organs of hoplonemertean and pilidia larvae point to two different paths in the evolutionary transformation of the ancestral apical organ. Ultrastructural and immunoreactive analyses of the apical organ of a hoplonemertean larva in the late rudiment and vermicular stages and the frontal organ of the adult worms identified common morphological and functional features. Thus, we hypothesize that the larval apical organ is modified during morphogenesis to form the adult frontal organ, which fulfills a sensory function in the hoplonemertean worm. This unique developmental trait distinguishes the Hoplonemertea from other nemertean groups.

## Background

The most prominent neural structure in spiralian larvae is the apical organ (sensu Wanninger [1]), which consists of an apical ciliary tuft and a receptor cell cluster [2]. Because the apical organ is composed of nervous system components (i.e., the bodies and/or processes of neurosecretory cells), it is considered a neurosecretory structure (see Wanninger [1] for a review) that plays an important role in environmental signal perception [3, 4]. The apical organ is proposed to fulfill critical functions during larval settlement [5, 6], metamorphosis [7–9], and locomotion [10]. Although the apical organ is completely reduced during metamorphosis [11], some of its nervous elements may remain and contribute to the formation of the “brain” in adult animals [12–15]. During the past 15 years, most studies on the apical organ have used confocal laser-scanning microscopy (CLSM) combined with various antibodies (see, e.g., Wanninger [1] and Richter et al. [16]). The ultrastructure of the apical organ has been studied in larvae of representatives of various groups of Spiralia, including: Platyhelminthes [17], Annelida [12, 18], Bryozoa [19–22], Phoronida [23–26], Brachiopoda [27], and Mollusca [3, 7, 28–33].

Within the Nemertea, another Spiralian clade, the apical organ has been identified in the larvae of its three main phylogenetic branches, but it has been well-studied only in the pilidia larvae of members of the Pilidiophora [14, 34–37]. Investigations of the structure of the apical organ in the planuliform larvae of Palaeonemertea and the decidula of Hoplonemertea have been conducted mainly by light microscopy [38], although some fragmentary data of this organ was obtained by transmission electron microscopy (TEM) [39] and CLSM [13]. Whereas pilidia larvae undergo a catastrophic metamorphosis, palaeonemertean planuliform larvae and hoplonemertean decidula larvae are gradually remodeled into the adult during metamorphosis, which is considered the plesiomorphic state among nemerteans [35]. The apical organ in planuliform larvae is believed to be reduced during metamorphosis, which is similar to the process observed in other invertebrate larvae [38, 40]. However, most adult hoplonemertean worms have a frontal organ located in the same position as the larval apical organ, and it probably performs a chemotactic function [41]. Some authors used the designation ‘apical organ’ to describe the frontal organ of adult nemerteans [42–44]. However, it is not known whether the frontal organ of the hoplonemerteans is a modified larval apical organ or a newly formed organ that occupies the site of the apical organ. The ultrastructure of the frontal organ, unlike that of other nemertean sense organs, has not been described to date, and its sensory function has not been unequivocally established.

In this work, we conducted ultrastructural and immunohistochemical studies on the larval apical and adult frontal organs of the hoplonemertean *Quasitetrastemma stimpsoni* (Chernyshev, 1992). Hoplonemertean larvae or decidulas are distinguished from planuliform larvae of the palaeonemerteans by a transitory epidermis consisting of larger cells, which is replaced during metamorphosis by the definitive epidermis formed from small cells [45–47]. Our study aimed at determining whether the apical organ of the decidula larvae corresponds to the apical organ of other spiralian larvae and whether it is related to the frontal organ of adult nemerteans.

## Results

### Definitions and characteristics of larval developmental stages

Larval development of *Q. stimpsoni* was examined on the light-optical level as described earlier [48]. According to this report, the length of the apical ciliary tuft gradually decreased during the post-fertilization period. The broad tuft was 90–120 μm long at 12–28 h post-fertilization (hpf), measured 50–60 μm at 48 hpf, and was reduced to 30–40 μm at 72 hpf when it consisted of a small number of long cilia. By 96 hpf, the apical tuft was reduced to one cilium of approximately 15–20 μm in length that was still detectable after settlement at 7–8 days post-fertilization (dpf). The neurogenesis of the serotonergic nervous system during early larval development (12, 16, 20, 25, and 50 hpf) in *Q. stimpsoni* has been described by Chernyshev and Magarlamov [13]. The authors observed that immediately after hatching, the early larva has an apical ganglion consisting of two apical and two subapical neurons. At the end of metamorphosis, the serotonergic neurons outlined the general structure of the nervous system of an adult nemertean.

The designations of *Q. stimpsoni* larval development stages proposed by Hiebert et al. [49, 50] were used in this study. Accordingly, larvae in the early rudiment stage at 36 hpf have a transitory (or provisory) epidermis composed of large multiciliated cells that they shed entirely by the end of the stage at 48 hpf. The nervous system consists of apical and subapical neurons, a caudal neuron (which is lost by 48 hpf), and the rudiments of brain lobes and lateral neurons that form the lateral nerve cords. By the late rudiment stage, larvae have well-developed lateral nerve cords that are connected in the posterior half of the larva. The brain lobes consist of two groups of cells connected by a ring of commissural tracts. By the vermicular stage, the ‘larvae’ are elongated and exhibit worm-like behaviors. Furthermore, the apical neurons are still visible but have lost the connection with the apical plate and brain rudiment. At this stage, the ‘larvae’ of *Q. stimpsoni* do not differ significantly from the juvenile worms that move by crawling after settling [48].

### Larvae in the early rudiment stage

The early rudiment stage was studied in larvae with the transitory epidermis at 36 hpf and the definitive epidermis at 48 hpf. The 36 hpf larvae were investigated using TEM only, and the observations were limited to significant features that are characteristic for this stage (see below). The 48 hpf larvae were examined using both TEM and CLSM.

**Fig. 1:**
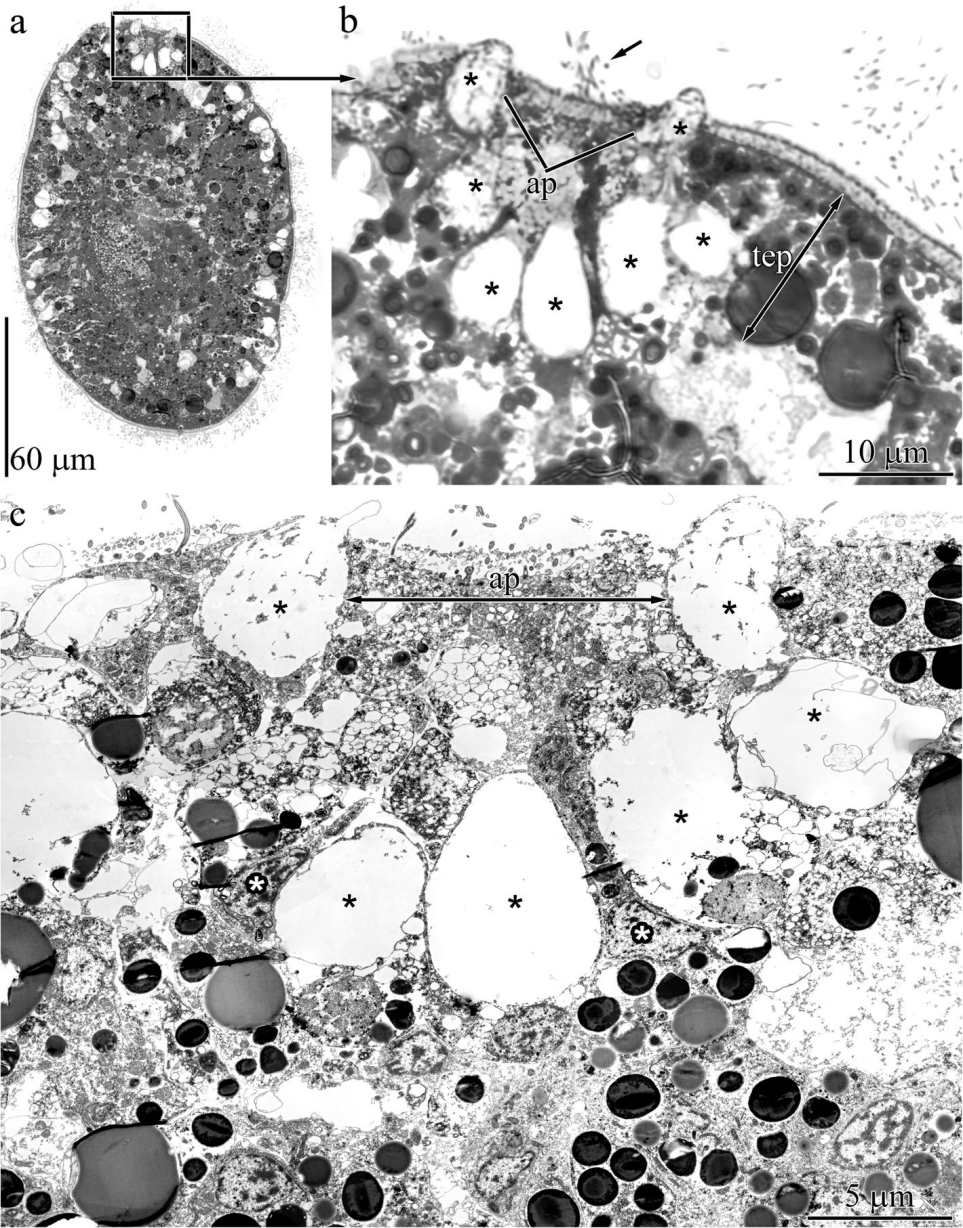
Light (**a**, **b**) and transmission electron (**c**) micrographs of sample sections, highlighting features of the epidermis in 36 h post-fertilization (hpf) larvae of *Quasitetrastemma stimpsoni*. **a** Panoramic view showing a larva. **b** The larval anterior tip with apical ciliary tuft (arrow) and apical plate (ap) positioned between the glandular cells (asterisks) of the frontal gland rudiment. **c** Panoramic view showing apical plate (ap) and the rudiment of frontal gland (black asterisks). The nuclei (white asterisks) of the multiciliated cells of the apical plate (ap). Abbreviations: ap–apical plate, tep–transitory epidermis

The larvae at the beginning of the early rudiment stage (36 hpf) are covered by a transitory epidermis with intercalating glandular cells of the definitive epithelium (Fig. 1a and b). The bodies of the elongated multiciliated cells of the apical plate (up to 30 μm in length) are positioned between the glandular cells of the frontal gland rudiment (Fig. 1c). The rudiment of the frontal gland consists of one glandular cell type (mucoid cells) (Fig. 1b and c).

**Fig. 2:**
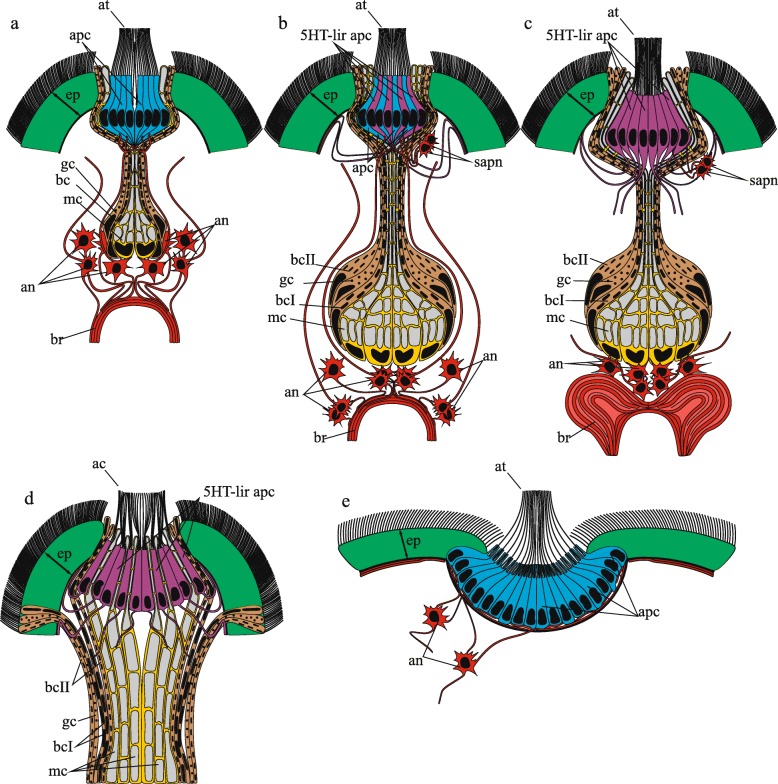
Schematic representation of the apical and frontal organs in Nemertea based on morphological data of neurogenesis and ultrastructure in the studied species. **a** The apical organ of 2 d post-fertilization (dpf) larva of *Quasitetrastemma stimpsoni* consists of an apical plate, frontal gland, and apical neurons. Frontal gland includes mucoid (mc), bacillary (bc), and granular (gc) cells. Apical neurons (an) are located beneath bodies of gland cells and connected with the brain rudiment (br) and the apical plate cells (apc) via neurites of apical neurons. **b** In the apical organ of 3–4 dpf larva of *Q. stimpsoni,* some apical plate cells possess 5-HT-like immunoreactivity (5-HT-lir apc). The frontal gland has four glandular cell types: mucoid cell (mc), granular cell (gc), bacillary cell type I (bcI), and bacillary cell type II (bcII). The pairs of subapical-plate neurons (sapn) are visible. **c** In the apical organ of 7 dpf larva of *Q. stimpsoni,* all apical plate cells are 5-HT-lir. The apical neurons (an) processes are resorbed. **d** The frontal organ of the adult worm of *Q. stimpsoni.* **e** The apical organ of *pilidium prorecurvatum* larva (the scheme was created according to [36]*.* Abbreviations: 5-HT-lir apc–5HT-like immunoreactive apical plate cell; ac–apical cilia; an–apical neuron; apc–non 5HT-like immunoreactive apical plate cell; at–apical tuft; br–brain rudiment; bc–bacillary cell; bcI–bacillary cell type I; bcII–bacillary cell type II; gc–granular cell; mc–mucoid cell; ep–epithelium; sapn–subapical plate neuron

**Fig. 3:**
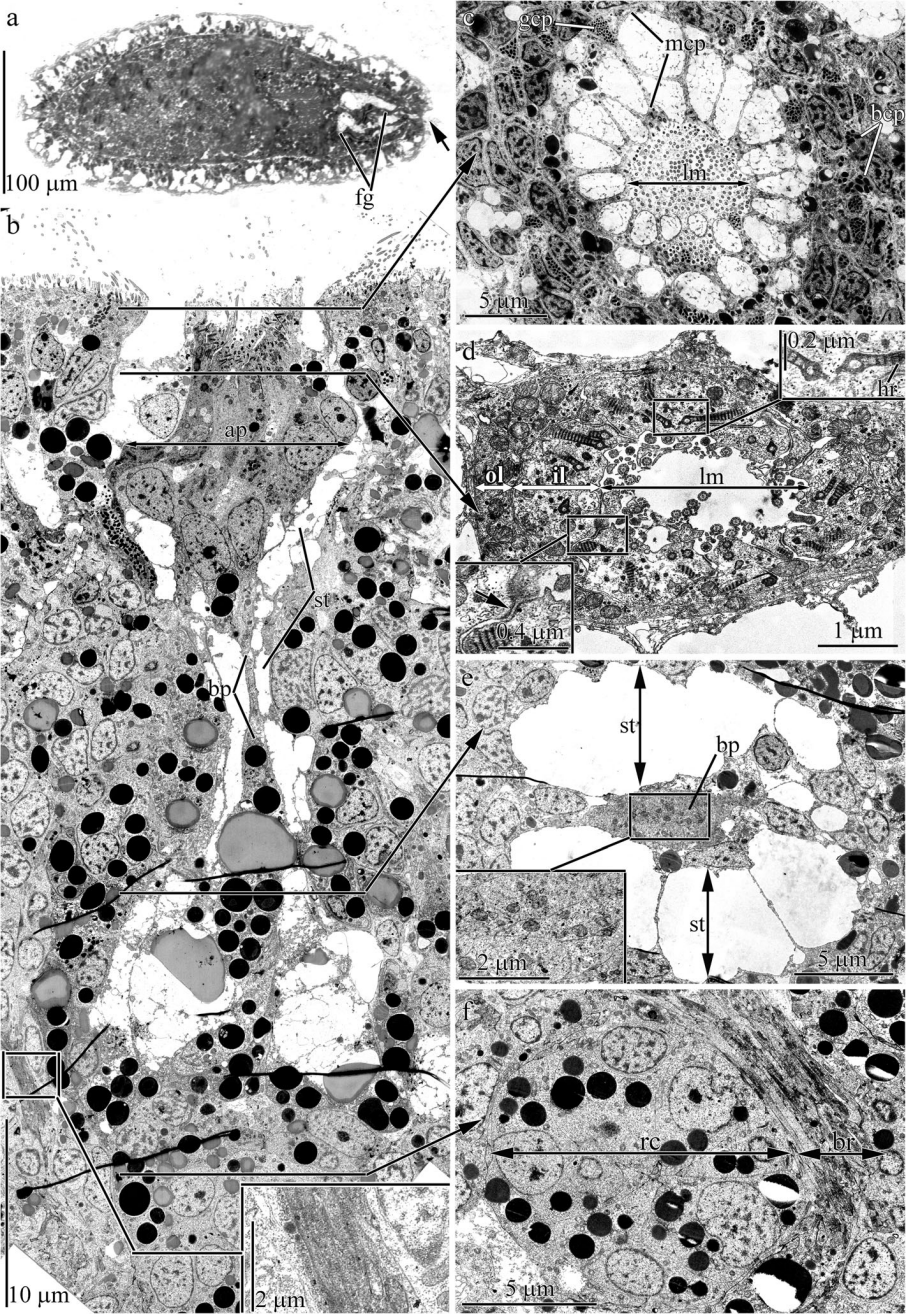
Light (**a**) and transmission electron (**b**-**f**) micrographs of sample sections highlighting the epidermis in 2 d post-fertilization (dpf) larvae of *Quasitetrastemma stimpsoni*. **a** Panoramic view showing larva with small pit (arrow) on the anterior end and a well-developed frontal gland (fg). **b** Panoramic view showing the longitudinal section through the apical organ. High-magnification inset shows the nerve tract. **c** The transversal section of the extreme anterior end of larva with a central lumen (lm) surrounded by the papillae of secretory cells. **d** Transversal section of the apical plate consisting of double cell rings with a centrally situated lumen (lm). High-magnification inset on the bottom left shows the apicolateral junction (arrow). High-magnification inset on the top right shows the cilia root of the apical plate cells. **e** Transversal section of secretory tract (st) with centrally situated basal processes (bp) of apical plate cells. High-magnification inset shows the basal processes of apical plate cells. **f** Transversal section through rudiments of the brain (b) and rhynchocoel (rc). Abbreviations: ap–apical plate; bcp–bacillary cell papilla; bp–basal process; br–brain rudiment; fg–frontal gland; gcp–granular cell papilla; hr–horizontal rootlet; il–inner layer of apical plate; lm–lumen of apical plate; mcp–mucoid cell papilla; ol–outer layer of apical plate; rc–rudiment of rhynchocoel; st–secretory tract

**Fig. 4:**
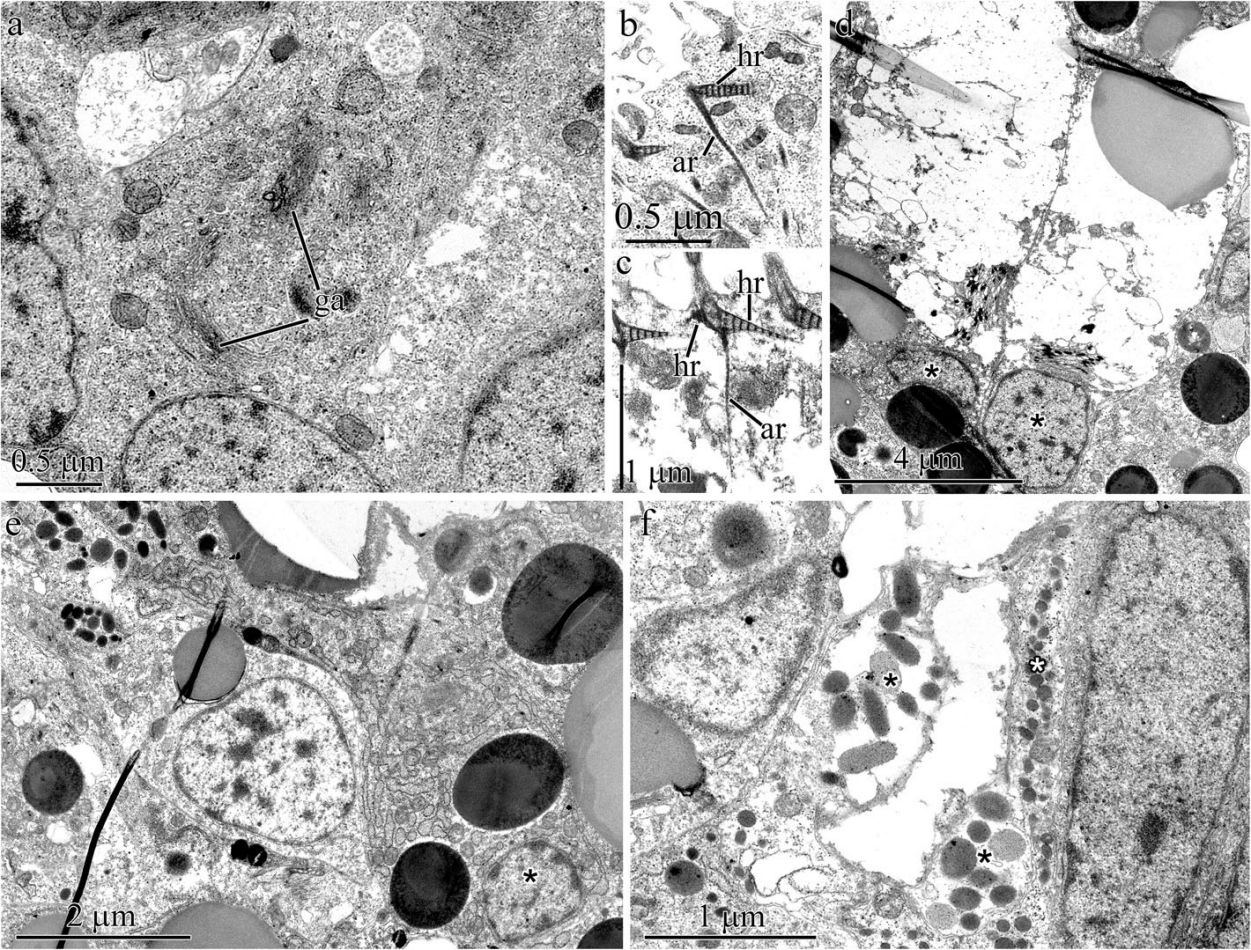
Transmission electron micrographs of epidermis in 2 d post-fertilization (dpf) larvae of *Quasitetrastemma stimpsoni*. **a** Central region of apical plate cells. **b** The cilia rootlets of apical plate cells. **c** Ciliary rootlets of cell of definitive epidermis. **d** The nuclei (asterisks) of mucoid cells. **e** The nucleus (asterisk) of a bacillary cell. **f** Extensions of bacillary (black asterisks) and granular (white asterisk) secretory cells. Abbreviations: ar–axial rootlet; hr–horizontal rootlet; ga–Golgi apparatus

At 2 dpf, the larvae are covered by a definitive epidermis (see Fig. 4a, d, and f in [46]). The apical pit (15–18 μm in diameter), which is associated with the well-formed ciliary tuft, occupies the anterior pole of the larva (Figs. 2a and 3a). The apical plate at the bottom of the apical pit is surrounded by the ducts of frontal gland cells. The apical plate, which features a narrow central lumen (2.5–3.5 μm in diameter and approximately 6 μm in depth), has a diameter of approximately 10 μm and a thickness of approximately 35 μm (Fig. 3b and c). In the transversal sections, the plate consists of an outer and an inner concentric cell layer (Fig. 3d). The inner layer consists of 6–8 cells with a prismatic shape in the transversal sections, which form the wall of the central lumen. The apical surface and the inner lumen surface are covered by numerous cilia and microvilli. The outer layer of the apical plate shows four flattened cells in the transversal sections (0.5–0.7 μm in thick, 2.4–3.1 μm in length). The cells’ apical surface possesses 5–12 cilia surrounded by microvilli (Fig. 3d). The basal processes of the apical plate cells extend proximally from the cell body (Fig. 3b). The basal processes of adjacent cells converge in the interior part of the larva (Fig. 3e). In contrast to other larval cells, both the inner and outer layer of cells of the apical plate have a denser fine-grained cytoplasm and a smaller number of yolk granules (Figs. 3b and 4a); and their mitochondria are more abundant in the apical region near the ciliary rootlets (Fig. 4b). Adjacent cells of the outer and inner rings are joined by api-colateral junctions that resemble zonulae adherentes (Fig. 3d). The ciliary rootlet structures of the apical plate cells and the multiciliated cells of the definitive epidermis are different. The cilia rootlets of the apical plate cells comprise one cross-striated horizontal rootlet (up to 1 μm in length) and one well-developed cross-striated axial rootlet (up to 3 μm in length) (Fig. 4b). Their basal body is extended by short stick-like structure (approximately 50 nm in length) – presumably a basal foot, located on the opposite side of the horizontal rootlet (Fig. 3d). The ciliary rootlet of the cells of the definitive epidermis consist of short (up to 0.2 μm in length) and long (up to 0.8 μm in length) cross-striated horizontal rootlet and one thin cross-striated axial rootlet (up to 1.2 μm in length) (Fig. 4c).

The frontal gland occupies much of the precerebral region of the larvae (Figs. 2a and 3b). Their cells are oriented with the apical end directed anteriorly. The bodies of the glandular cells lie at a depth of 40 to 50 μm from the apical end of the larva (Fig. 3b) and are in proximity to the developing brain and the rhynchocoel rudiment (Fig. 3f). There are three glandular cell types in the frontal gland: mucoid, bacillary, and granular cells. The voluminous mucoid cells form a major portion of the frontal gland (Fig. 3a and b). They are filled with irregular granules with a diameter of up to 5 μm (Fig. 4d). Each granule is loosely packed with fibrillary material. The mucoid cell bodies extend to the epithelial surface via a cell neck (Fig. 3b), which converge to form a wide secretory tract that goes around the apical plate and extends to the larval surface (Fig. 3b, c, and e). The end of each cell neck expands apically to form a globular papilla that reaches approximately 4–6 μm above the epithelium surface. The apical plate is densely surrounded by the mucoid cell papillae; the papilla number varies between 20 and 25 (Fig. 3c). The ultrastructure of the bacillary and granular cells is similar and distinguished only by the shape and content of the secretory granules. Their bodies are located outside of the mucoid cell bodies (Fig. 3b), and their necks and papillae are filled with many granules (Fig. 4f). The bacillary cell granules are rod-shaped (0.21 ± 0.04 μm wide, *n* = 33; 0.6 ± 0.11 μm long, n = 33) and filled with homogeneous material of moderate to high electron density (Fig. 4e and f). The granular cells have spherical secretory granules (0.1 ± 0.02 μm in diameter; *n* = 36) containing homogeneous, moderately electron-dense material. The necks of the bacillary and granular cells expand apically to form a bulbous protrusion on the outside along the papillae of the mucoid cells (Fig. 3c). The number of protrusions varies from 14 to 15 for bacillary cells and from 3 to 5 for granular cells. Well-developed nerve tracts are found in the apical end of the larvae near the frontal gland and apical plate (Fig. 3b).

### Larvae in the late rudiment stage

**Fig. 5:**
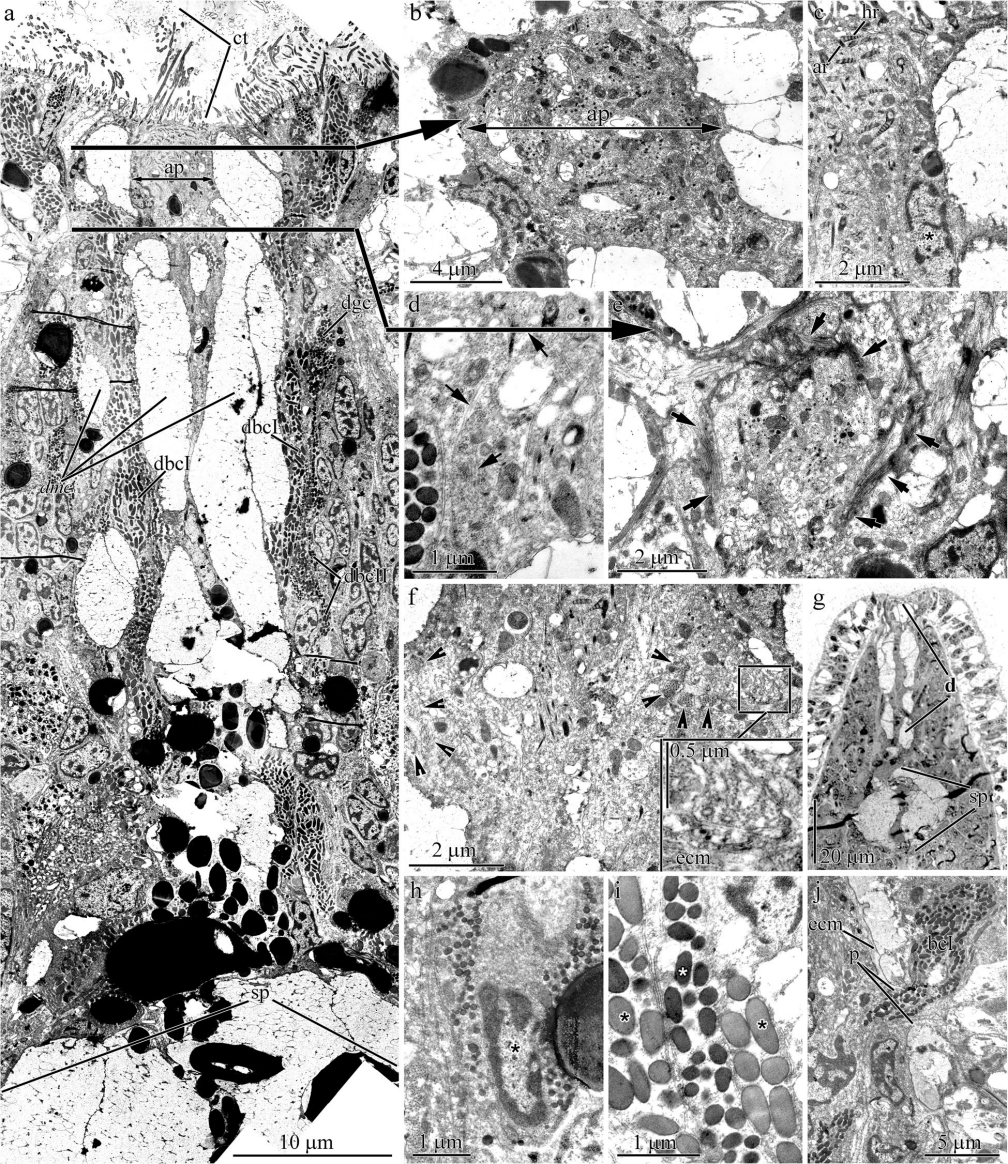
Transmission electron (**a**-**f**, **h**-**j**) and light (**g**) micrographs of sample sections of the epidermis in 3–4 d post-fertilization (dpf) larvae of *Quasitetrastemma stimpsoni*. **a** Panoramic view showing the longitudinal section through the apical organ. **b** Transversal section of the apical plate (ap). **c** Longitudinal section of multiciliated cells of the apical plate (asterisk). **d** Middle region of multiciliated cells of the apical plate with longitudinal arrays of microtubules (arrows). **e** Transversal section through the sphincter (arrows) located in the basal half of the apical plate. **f** Longitudinal section of the basal half of the apical plate. Arrowheads point to circular musculature rows of the sphincter. High-magnification inset shows the basal extensions formed by apical plate cells which are attached to sphincter wall. **g** Panoramic view showing the anterior end of the larva with secretory part (sp) and ducts (**d**) of frontal gland. **h** Granular cell (asterisk) of frontal gland. **i** Bacillary type I (black asterisks) and type II (white asterisk) cell extensions. **j** Secretory extensions of bacillary type I cells extend through pore (p) in extracellular matrix (ecm). Abbreviations: ap–apical plate; ar–axial rootlet; ct–ciliary tuft; d–ducts of frontal gland; dbcI–frontal gland ducts of bacillary cell type I; dbcII–frontal gland ducts of bacillary cell type II; dgc–frontal gland ducts of granular cell; dmc–frontal gland ducts of mucoid cell; ecm–extracellular matrix; hr–horizontal rootlet; p–pore in extracellular matrix; sp–secretory part of frontal gland

On the extreme anterior end of the larvae, the apical pit (approximately 18 μm in diameter) associated with the ciliary tuft is clearly visible (Fig. 5a). Its structure is similar to that at the early rudiment stage (50 hpf). The bottom of the pit consists of an apical plate surrounded by the ducts of the frontal gland (Fig. 2b). In the transversal sections, the apical plate consists of 8 to 10 centrally situated cells with a prismatic shape, which are surrounded by 4 flattened cells (Fig. 5b). There is no lumen in the apical plate. In the longitudinal section, the epithelium is formed by the slender apical plate cells (Fig. 5a and c). The ultrastructure of the larval apical plate cells in the early rudiment stage (Figs. 3b, 4a, and b) and late rudiment stage (Fig. 5a, c, and d) are similar. Adjacent to the apical plate is a sphincter-like structure, which consists of 3 to 4 rows of muscle cells surrounded by connective tissue (Fig. 5e and f). The basal extensions formed by apical plate cells are either attached to the wall of the sphincter-like structure or pass deep into the larva through an opening of the sphincter-like structure (Fig. 5f).

The frontal gland occupying the anterior end of the late rudiment stage larva differ from that of the early rudiment stage larva (50 hpf) only by possessing a more expanded spherical-shaped secretory portion and broader excretory elements (ducts) (Figs. 2b and 5g). The larval mucoid cells in the early and late rudiment stage share a similar ultrastructure, whereas the granular and bacillary cells differ in the shape and content of the secretory granules. Granular cells are filled with rounded granules (diameter of 0.14 ± 0.02 μm, *n* = 22) containing homogeneous, moderately electron-dense material (Fig. 5h). There are type I and II bacillary cells. Secretory granules of bacillary cell type I have an oval or stick-like shape with a length of 0.9 ± 0.18 μm (n = 22) and a diameter of 0.34 ± 0.04 μm (*n* = 20) (Fig. 5i). Bacillary cell type II granules (0.47 ± 0.05 μm length, n = 22; 0.26 ± 0.04 μm diameter, n = 22) have dark content (Fig. 5i). Ducts of the mucoid, granular, and bacillary type II cells open into the anterior pit (Fig. 5a), whereas bacillary type I cells open into the anterior tip (Fig. 5a) and at various points along the length of the frontal gland (Fig. 5j).

### Adult worm

**Fig. 6:**
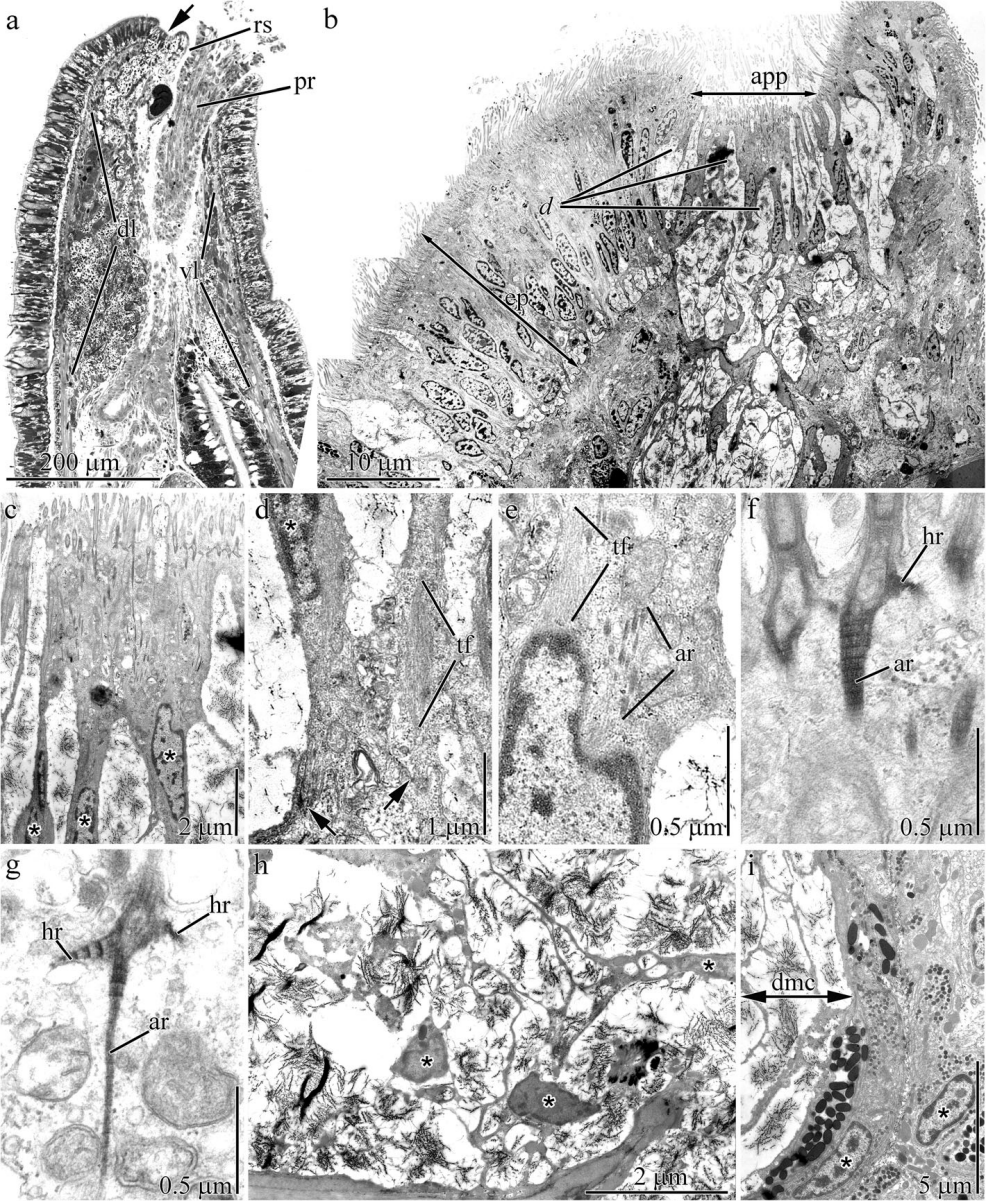
Light (**a**) and transmission electron (**b**-**i**) micrographs of sample sections of epidermis in adult worms of *Quasitetrastemma stimpsoni*. **a** Panoramic view of the longitudinal section of the anterior end of the worm with an apical pit (arrowhead). **b** Panoramic view of longitudinal section of extremely anterior end. **c** Multiciliated cells (asterisks) located between ducts of the frontal gland. **d** Basal region of the multiciliated cells (asterisk) of the apical pit attached to the extracellular matrix (arrows). **e** Perinuclear cytoplasm of multiciliated cells of the apical pit. **f** Ciliary rootlets comprising horizontal (hr) and axial (ar) rootlet of multiciliated cell of the apical pit. **g** Ciliary rootlets comprising two horizontal (hr) and one axial (ar) rootlet of multiciliated cells of the epidermis. **h** Bodies of mucoid cells (asterisks). **i** Periphery of the secretory portion of the frontal gland with nuclei of granular gland cells (asterisks). Abbreviations: app–apical pit; ar–axial rootlet; d–ducts of frontal glands; dl–dorsal lobe of frontal gland; dmc–ducts of mucoid cells; ep–epidermis; hr–horizontal rootlet; pr–proboscis; rs–rhynchostome; tf–tonofilaments; vl–ventral lobe of frontal gland

At the anterior end of the adult worm (approximately 30 mm in total length), dorsal to the rhynchostome, is a small apical pit (approximately 18 μm diameter) (Fig. 6a) with openings for the frontal gland ducts (Figs. 2d and 6b). Between the ducts, slender multiciliated cells are present as single cells or small cell clusters of 2 to 4 cells (Fig. 6c). These cells are anchored to the subepithelial extracellular matrix (ECM) by a basal extension (Fig. 6d). Tonofilament bands (10–18 nm thick) occupy the perinuclear cytoplasm (Fig. 6d and e).

While apical pit cells and epidermal multiciliated cells both possess ciliary rootlets, they differ in the number and morphology of their rootlets. The ciliary rootlets of the apical pit multiciliated cells comprise one short (approximately 0.14 μm) cross-striated horizontal rootlet (Fig. 6e) and one well-developed axial rootlet (up to 6 μm in length) (Fig. 6e and f). Adjacent axial rootlets converge and end in the perinuclear region of the cell (Fig. 6e). The ciliary rootlets of epidermal multiciliated cells have one small (approximately 0.15 μm in length) and one long (approximately 0.4 μm in length) cross-striated horizontal rootlet and one thin cross-striated axial rootlet (up to 2.3 μm in length) (Fig. 6g).

**Fig. 7:**
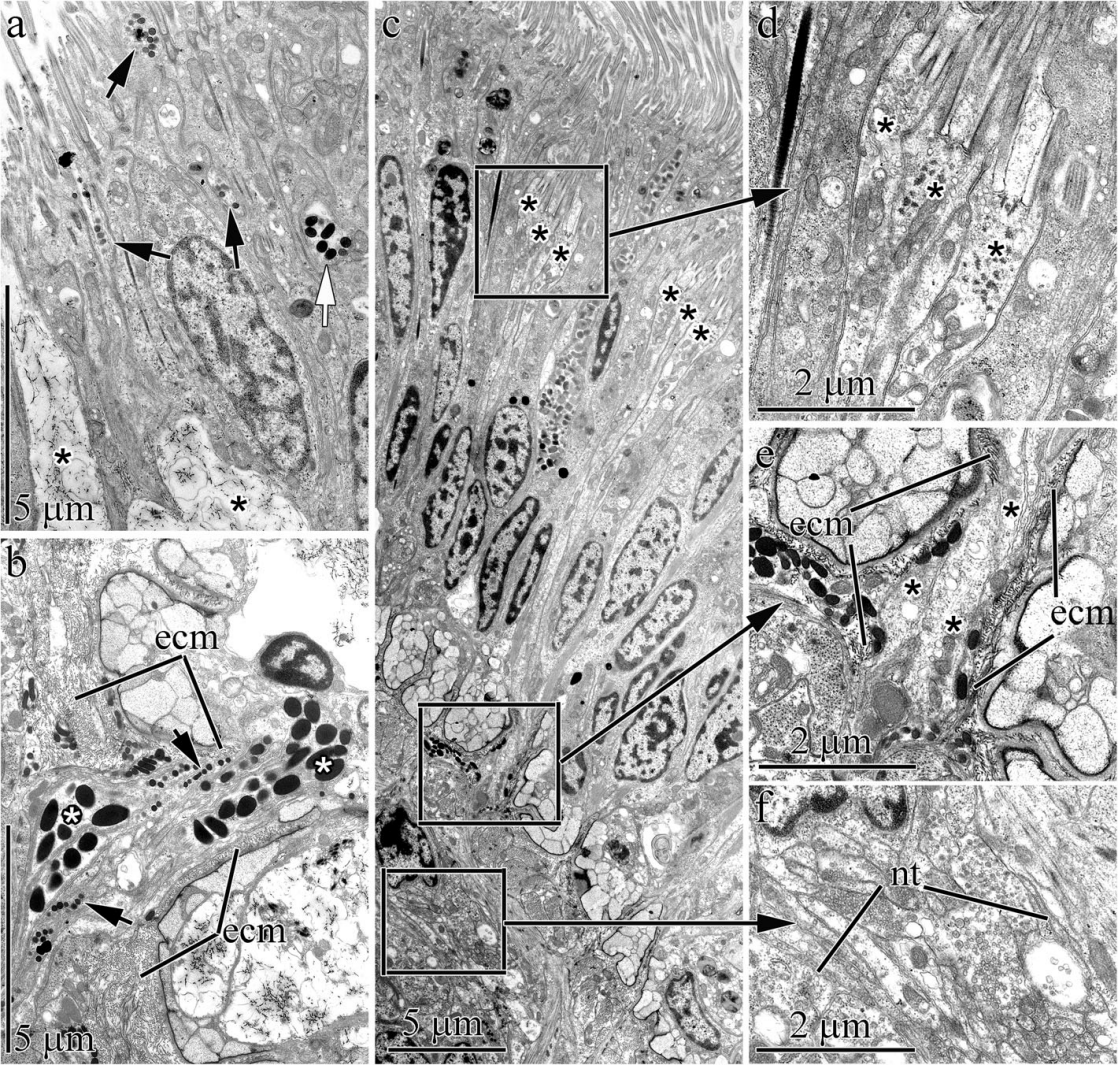
Transmission electron micrographs of the epidermis in adult worms of *Quasitetrastemma stimpsoni*. **a** Ducts of the mucoid (asterisks), granular (black arrowheads), and bacillary type II (white arrowhead) glandular cells. **b** Secretory extensions of bacillary type I cells (white asterisks) and granular cells (arrowheads) extend through a pore in the extracellular matrix (ecm). **c** Panoramic view shows the dorsal epidermis near the apical pit with two groups of sensory cells (asterisks). **d** Group of sensory cells (asterisks). **e** Axons (asterisks) of sensory cells extend through pores in the subepithelial extracellular matrix (ecm). **f** Subepithelial nerve tract. Abbreviations: ecm –extracellular matrix; nt–nerve tract

The frontal gland is located deep in the anterior end of adult worm occupying the entire precerebral region (Fig. 6a). The gland forms a large dorsal and small ventral lobe. There are no differences in the cellular composition of the lobes. The mucoid cell bodies are associated and form the basis of the frontal gland in adult worms (Fig. 6h). The type I and II bacillary cells and granular cells are located along the frontal gland periphery (Fig. 6i). Granular and bacillary I and II type cells open into the apical pit (Fig. 7a) or at various points along the length of the frontal gland (Fig. 7b). The wide mucoid cell ducts open only into the apical pit (Fig. 6b).

Two groups of sensory cells are present on the dorsal side near the apical pit (Fig. 7c). These sensory cells carry a small perikaryon and possess proximally extended axons and distally extended dendrites (Fig. 7d). The dendrites extend to the apical surface to form the receptor process, which consists of a central cilium enclosed by a ring of microvilli. Axons of the sensory cells extend through pores across the subepithelial ECM (Fig. 7e) and end in the subepithelial nerve tracts (Fig. 7f).

### Serotonin-like immunoreactive components in the larvae and adults of *Q. stimpsoni*

**Fig. 8:**
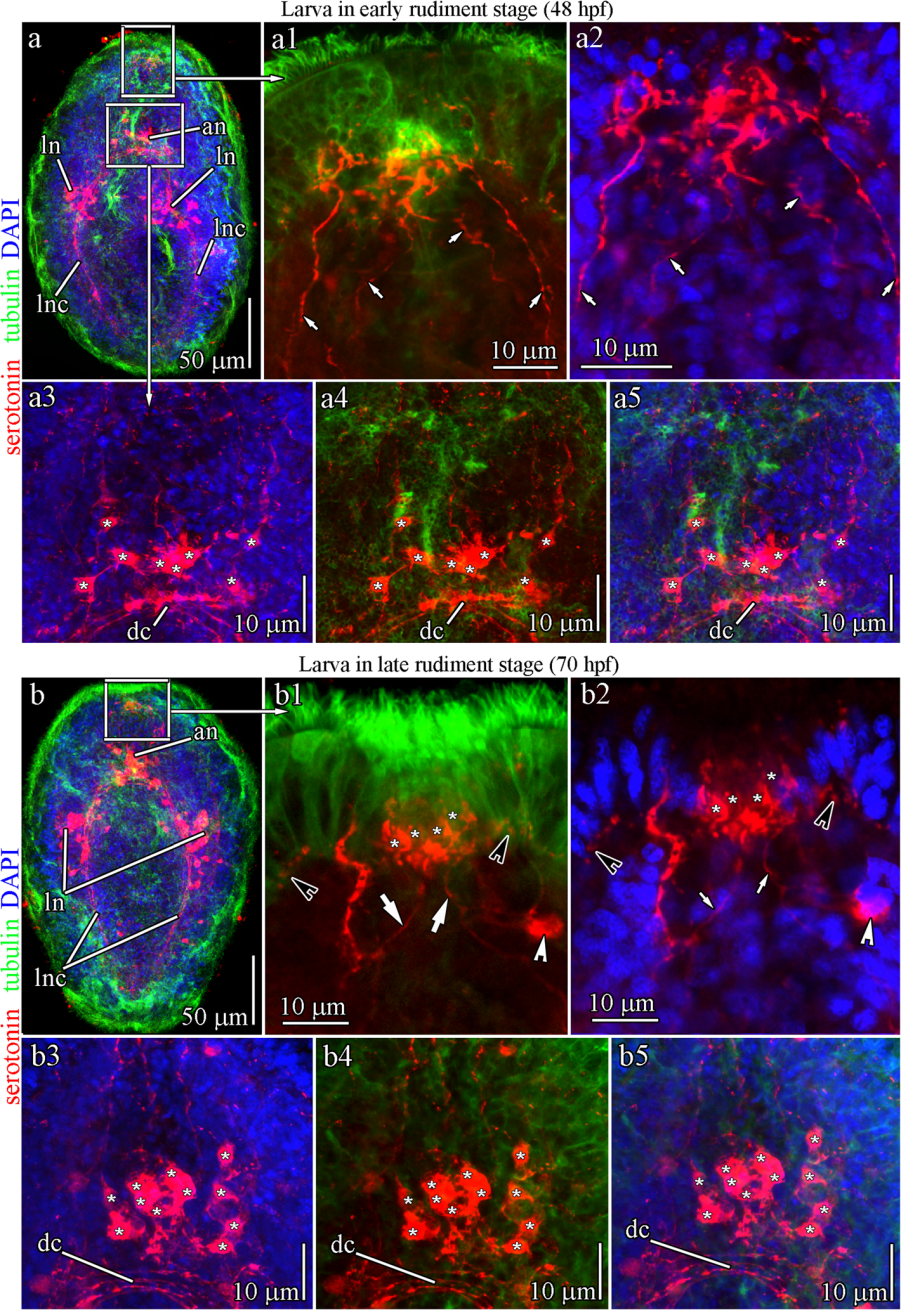
5-HT-like immunoreactivity (5-HT-lir) in the early and late rudiment stage larvae of *Quasitetrastemma stimpsoni*. Red - 5-HT-lir; green–cilia, acetylated tubulin immunoreactivity; blue—nuclei, DAPI. The apical pole is always upward; side view. **a** General view of 5-HT-lir in early rudiment stage larva, 48 h post-fertilization (hpf), with apical neurons (an) and paired lateral neurons (ln) located along the lateral nerve cord (lnc). **a1-a2** High-magnification inset shows apical plate cells innervated by neurites (arrows) of apical neurons. **a3-a5** High-magnification inset shows the apical neurons (asterisks) localized near dorsal commissural tract (dc). **b** General view of 5-HT-lir in late rudiment stage larva (70 hpf). **b1-b2** 5-HT-lir cells (asterisks) in the apical plate are innervated by neurites of apical neurons (arrows) and form a single axon (black arrowhead) projecting onto the subepithelial nerve plexus. Localized under the apical plate is a pair of 5-HT-lir subapical neurons (white arrowhead). **b3-b5** High-magnification inset of apical neurons (asterisks) localized near dorsal commissural tract (dc). Abbreviations: an–apical neuron; dc–dorsal commissural tract; ln–lateral neuron; lnc–lateral nerve cord

**Fig. 9:**
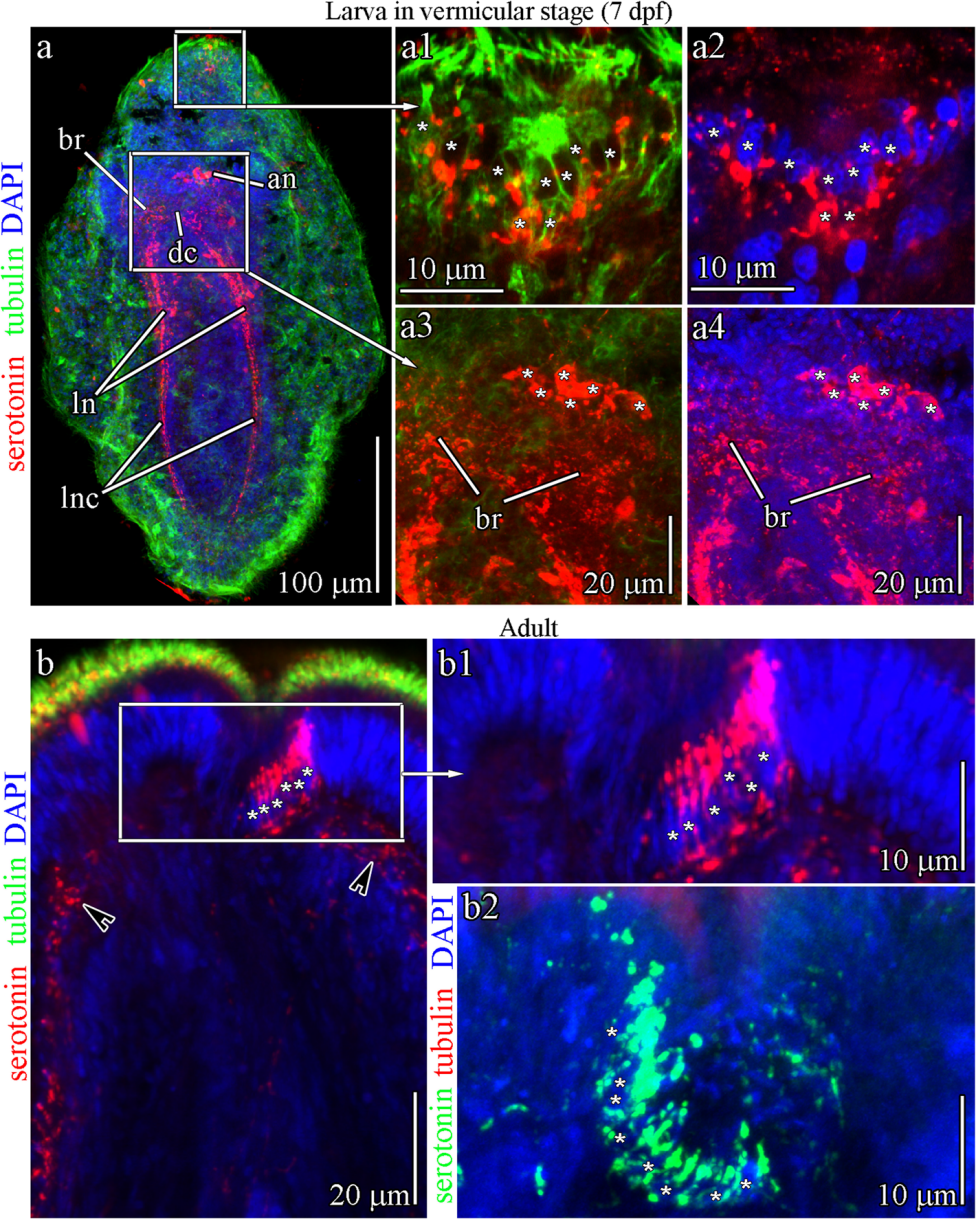
5-HT-like immunoreactivity (5-HT-lir) in the vermicular stage larva and adult worm of *Quasitetrastemma stimpsoni*. In **a-a4**, **b** and **b1**: red - 5-HT-lir, green–cilia (acetylated tubulin immunoreactivity), blue—nuclei (DAPI). In **b2**: red–cilia (acetylated tubulin immunoreactivity), green–5-HT-lir, blue—nuclei (DAPI). The apical pole is always upward; side view. **a** General view of 5-HT-lir in the vermicular stage larva, 7 d post-fertilization (dpf) with paired brain lobes (br) connected by dorsal commissural tract (dc) and extending lateral nerve cords (lnc). **a1**-**a2** Apical organ cells, 5-HT-lir positive (asterisks). **a3**-**a4** High-magnification inset shows resorbed apical neurons (asterisks). **b** General view of 5-HT-lir cells (asterisks) in the frontal organ of the adult worm. Tiny processes (arrowhead) of 5-HT-lir cells running from the organ to the left and right sides of the nemertean body wall. **b1**-**b2** High-magnification inset shows 5-HT-lir cells (asterisks) in the frontal organ. Abbreviations: an–apical neuron; br–brain rudiment; dc–dorsal commissural tract; ln–lateral neuron; lnc–lateral nerve cord

The larvae and adult worms of *Q. stimpsoni* contain serotonin (5-hydroxytryptamine)-like immunoreactive (5-HT-lir) components (Figs. 2a-d, 8a, b, Fig. 9a, and b). The 5-HT-lir signals are associated with the apical neurons and paired lateral neurons that are aligned along the lateral nerve cord (Figs. 8a, b, and 9a). In the early rudiment stage (48 hpf), neurites of apical neurons reach the apical plate and abundantly innervate the plate epithelium (Fig. 8a1 and a2). The larval apical organ has eight neurons (Fig. 8a3-a5), but the apical plate cells do not contain serotonin at 48 hpf. In the *Q. stimpsoni* larva at 70 hpf, the innervation of the apical plate by 5-HT-lir neurites becomes more complex as they form dense aggregates around the apical plate (Fig. 8b-b2). The 5-HT-lir sensory cells in the apical plate appear during this stage. In addition, paired neurons with 5-HT-lir elements that are laterally located in proximity to the apical plate (i.e., subapical-plate neurons) are visible (Fig. 8b1 and b2). At 70 hpf, the larval apical plate is externally innervated from below the subapical-plate and possesses apical neurons that are associated with 5-HT-lir signals. The larval apical plate cells form single axons projecting to the subepithelial nerve plexus (Fig. 8b1 and b2). At this stage, 11 apical neurons are detectable (Fig. 8b3-b5). In the 7 dpf ‘larva’ (free-swimming juvenile), the 5-HT-lir signals are associated with paired brain lobes connected by the dorsal commissural tract and the lateral nerve cords that extend from each brain lobe (Figs. 2c and 9a). At this stage, the apical neurons are still visible and in the process of resorption (Fig. 9a3 and a4); their number is reduced to six cells. In high magnification images, serotonin is detectable in the apical plate even without a connection to the apical neurons (Fig. 9a1 and a2).

The frontal sense organ of adult hoplonemertean *Q. stimpsoni* is positive for 5-HT-lir signals (Figs. 2d and 9b). The sense organ consists of a minimum of 8–10 5-HT-lir cells (supported by DAPI staining), which are densely packed to form a roundish pit (Fig. 9b). The 5-HT-lir cells possess tiny processes that extend laterally from the organ involved in the formation of the subepithelial nerve plexus. Two anti-5-HT-lir antibody preparations derived from two different clones had the same immunostaining pattern, demonstrating that the frontal organ of the adult *Q. stimpsoni* is the 5-HT-lir-containing sense organ (Fig. 9b1 and b2). Thus, it appears that the frontal sense organ is connected with the brain lobes via the lateral processes of its sensory cells to function as a component of the peripheral nervous system.

## Discussion

### Apical organs of the palaeo- and hoplonemertean larvae

According to recent phylogenetic analyses [51, 52], Palaeonemertea is a sister group (class – see [53]) to other nemerteans (or Neonemertea [54]). The planuliform larva of palaeonemerteans resembles a decidula in appearance [38, 40, 55] despite the lack of a transitory epidermis composed of large multiciliated cells [35]. The larvae of basal Palaeonemertea *Carinoma tremaphoros* have a vestigial prototroch, which are not found in larvae of hetero- and hoplonemerteans [55]. The analysis of the apical organ structure of palaeonemertean larvae, which is mainly limited to light-optical data, revealed a well-developed apical plate with an apical tuft. A study of the larvae (1 dpf) of the palaeonemertean *Carinina ochracea* identified two types of 5-HT-lir flask-shaped cells, one in the apical and median position and another in the medio-ventral position [56]. Available data do not allow a comparison of the apical organ of the paleonemertean larvae with those of the Pilidiophora and Hoplonemertea larvae.

The larvae of hoplonemerteans share a similar morphology and have an apical organ and a provisory epithelium, which is gradually displaced by the cells of the definitive epidermis [45, 46, 49, 50]. The suggested name given to this larval type is decidula [57]. During the early development of the decidula, the neuronal organization of the apical organ includes a few flask-shaped cells, each forming a single dendrite projecting into the vicinity of the apical pit [13, 14, 50]. Two pairs of 5-HT-lir neurons with a flask-shaped outline have been detected in the anterior end of decidula larvae of *Pantinonemertes californiensis* [50] and *Quasitetrastemma stimpsoni* [13], i.e., one pair of apical neurons and a second pair of ventrally located subapical neurons. The apical and subapical neurons are connected by two additional apical neurons, and we propose that these six neurons, i.e., two apical neurons, two subapical neurons, and two additional neurons, form an interconnected complex, which appears to be a component of the larval apical organ. The fate of the apical organ neurons in hoplonemertean species is not clear; some authors suggested that these neurons are completely resorbed during metamorphosis [50, 56], whereas other reports indicated that some apical neurons persist through the metamorphosis as components of the brain ring [13, 14, 58]. This study on swimming larvae in the vermicular stage (7 dpf) revealed six apical neurons but their connections to the brain ring and the apical plate cells were fully resorbed.

Only the apical organ of the hoplonemertean species *Carcinonemertes epialti* Coe 1902, a parasite of brachyuran crabs, has been examined by electron microscopy [39]. The apical organs of *C. epialti* and *Q. stimpsoni* larvae have a highly similar morphology based on a glandular sensory structure that consists of an apical plate, apical neurons, and frontal gland. The larval frontal gland in both hoplonemertean species typically consists of mucoid cells that directly surround the apical plate (Fig. 3b and c, see Fig. 21 in [39]). In addition to the mucoid cells, the frontal gland in both species also possesses granular cells, whereas only the larval frontal gland in *Q. stimpsoni* has bacillary cells. Interestingly, the ciliary rootlet morphology differs between the two hoplonemertean larvae: *C. epialti* possesses one nonstriated, oblique rootlet, whereas *Q. stimpsoni* has cross-striated horizontal and axial rootlets.

### Apical organs of the pilidiophoran larvae

**Fig. 10:**
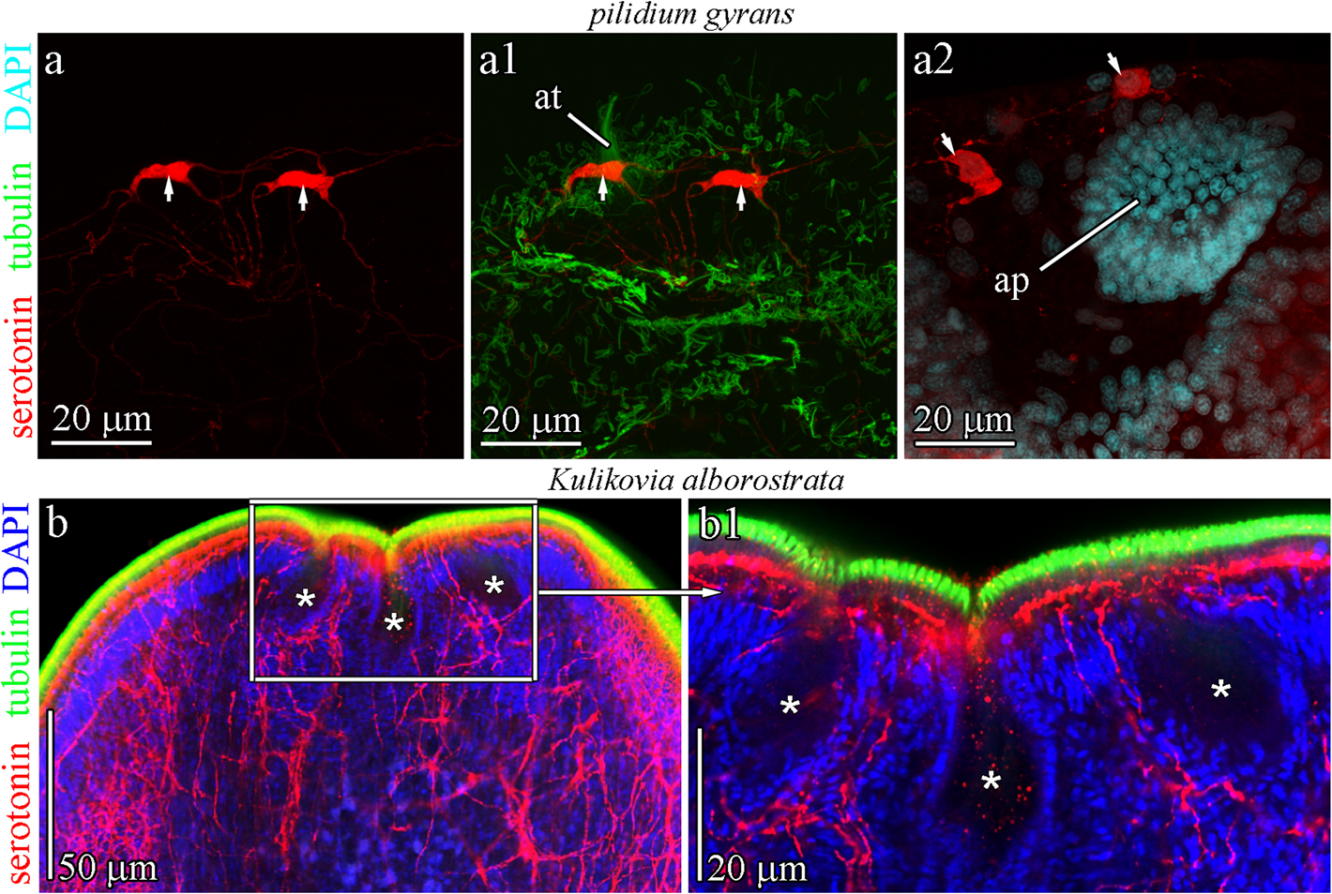
5-HT-like immunoreactivity (5-HT-lir) in the *pilidium gyrans* larvae and adult of *Kulikovia alborostrata* (Heteronemertea). Red - 5-HT-lir, green–cilia (acetylated tubulin immunoreactivity), blue—nuclei (DAPI). The apical pole is always upward; side view. **a-a2** Apical organ with two 5-HT-lir neurons (arrows) and apical tuft (at) in the *pilidium gyrans* larva. Tiny processes of 5-HT-lir cells surround an apical plate (ap). **b** General view of three frontal organs (asterisks) in *K. alborostrata*. 5-HT-lir neurites of subepithelial nerve plexus form dense aggregations beneath the frontal organs. **b1** High-magnification inset of frontal organs (asterisks) in *K. alborostrata*. Abbreviations: an–apical neuron; at–apical tuft

The apical organ morphology differs significantly between the hoplonemertean larvae and the heteronemertean pilidia (Fig. 2a-c and e). The pilidium possesses a simple apical organ that consists of an apical plate surrounded by a cup-shaped plexus of neuron processes (without neuron bodies) (Fig. 2e) [35–37]. In the examined species, two non-flask-shaped 5HT-lir neurons are associated with the apical plate of the pilidium larva (Fig. 10a) [35, 36]. The neurites of these cells contribute to the epidermal cup-shaped plexus (Fig. 10a and a1) [58]. No gland was observed in the pilidium apical organ. Similar to that in the *Q. stimpsoni* larva, the cells of the apical plate of the pilidium differ strongly from the other epithelial cells with a more slender shape and denser cytoplasm [36]. However, the cell numbers in the apical plate differ significantly between the pilidium and the decidula larvae. According to CLSM data with DAPI, the cell numbers in the pilidium of *Lineus albocinctus* (see Fig. 2c in [37]) and the *pilidium gyrans* larva (Fig. 10a2) are approximately 100, whereas the cell number in the lecithotrophic pilidium of *Micrura* sp. “dark” (see Fig. 5b in [59]), is typically above 400. Transversal sections of the apical plate of the *Q. stimpsoni* larvae in the rudiment stage consist of 8–10 centrally situated cells with prismatic shapes, which are surrounded by four flattened cells (Figs. 3d and 5b). The greater number of cells in the apical organ of pilidia can be interpreted as stemming from the fact that the apical organ is more long-lived than that in hoplonemertean larvae. Each cell in the apical plate of the pilidium larva bears a single [35, 36, 60] or several [34] cilia surrounded by a microvilli collar, which is absent in the hoplonemertean larvae. Interestingly, during rapid metamorphosis, the apical organ and the rest of the pilidial body of the pilidium are typically consumed (eaten, devoured, swallowed) or (in case of Hubrechtiiformes) discarded. In contrast, the apical organ of the hoplonemertean larvae stays after the replacement of the transitory epidermis by the definitive epidermis in early larval development.

Significant differences in the structure of the apical organs of hoplonemertean and pilidia larvae point to different evolutionary paths of the ancestral apical organ in Hoplonemertea and Pilidiophora. Flask-shaped serotonergic cells of the larval apical organ, considered plesiomorphic for the spiralians [1], are not found in pilidial larvae. Whether the morphologically simple apical organ of pilidiophoran nemerteans is a plesiomorphic or derived (as is in some polychaets [60]) structure remains to be tested.

### Apical organs of the hoplonemertean and other spiralian larvae

A comparison of the ultrastructure of apical organs of decidula and representatives of other Spiralia groups shows that only the Müller’s and Götte’s larvae of the polycladid flatworms [17, 61] have an apical organ consisting of an apical plate associated with subepithelial gland cells. In the hoplonemertean larvae (i.e., *Q. stimpsoni* (current study) and *Carcinonemertes epialti* [39]) and Müller’s larva of *Pseudoceros canadensis* [17], each gland cell body forms long ducts that are extended to an apical end of the larva; the ducts densely surround the apical plate and extend anteriorly to form bulbous protrusions at the larval surface. However, there are some differences in the ultrastructural organization between the apical organs of Müller’s and the hoplonemertean larvae. The apical plate of the Müller’s larva consists of four to five monociliated flask-shaped cells. The expanded cell bodies of the apical plate cells lie on the top of the brain rudiment; from each body, a long thin process extends toward the anterior end of the larva. In the hoplonemertean larva, the apical plate consists of 10 to 12 multiciliated slender and columnar cells, the bodies of which lie beneath the epidermis. Only two glandular cells have been identified in the apical organ of Müller’s larva [17]; the bodies of the glandular cells are located below the dorsal surface of the brain, and their necks are bifurcated to form up to 12 apical bulbous protrusions at the larval surface. The frontal gland of the *Q. stimpsoni* larvae consists of numerous glandular cells, each of which forms a non-bifurcated neck.

Hoplonemertean and polycladid flatworm larvae share features of their immunoreactivity profiles of the apical plate cells and their relationship with the central nervous system [17, 62, 63]. The apical plates of the larvae of both the polycladid *Maritigrella crozieri* [63] and the hoplonemertean *Q. stimpsoni* (current research) have positive immunoreactivity for neurotransmitters (5HT and FMRF-amide in *M. crozieri* and 5HT in *Q. stimpsoni*). The ciliated cells of the tuft in *M. crozieri* larva and *Q. stimpsoni* larva at the rudiment stage are linked to the central nervous system by “intermediate” neurons. In the polycladid larvae, the “intermediate” neurons consist of large clusters of neurites, whereas in *Q. stimpsoni* larvae of apical neurons. The apical plate cells of the polycladid larvae do not form axon projections [64], but the apical plate cells of the *Q. stimpsoni* larvae form axon projections into the subepithelial nerve plexus during the late rudiment stage (Fig. 8b2). Intraepidermal, flask-shaped 5-HT-lir cells have not been detected in the apical organ of the *M. crozieri* larva. However, between one and four flask-shaped 5-HT-lir cells have been detected in the decidula larvae (current study and [14]).

The presence of a small number of flask-shaped receptor cells in the apical organ is a basal feature in spiralian protostomes [1, 65], especially in Annelida, Mollusca, and Brachiopoda [11, 14, 33, 66]. The presence of a flask-shaped 5-HT-lir monopolar perikaryon in the apical organ most likely constitutes a lophotrochozoan apomorphy, and their small number may be a basal trait within Lophotrochozoa [67]. During the early larval stages in the palaeo- and hoplonemerteans, the number of flask-shaped neurons in the apical organ varies between one and four (for comparison, the phoronid apical organ contains more than 40 5-HT-lir flask-shaped cells [67]). The absence of flask-shaped cells in the apical organ of the polycladid flatworm larvae can be either the plesiomorphic or a derived condition [63]. Based on current spiralian phylogenies, the similarity between the apical organs of the polycladid larvae and the decidula, is the likely result of convergence.

### Apical and frontal organs in hoplonemerteans: are they developmentally connected?

Non-homologous structures called the “frontal organs” exist in various invertebrates, including the Acoelomorphs [68–72], phoronid larva [26], and decapod nauplii [73]. In the phoronids, the frontal organ performs an attachment function and is not associated with the apical organ of the larvae [74]. Typically, the frontal organ of hoplonemerteans is described as a flask-shaped protrusible pit opening at the anterior tip of the head above the rhynchopore associated with the cephalic gland [41]. The hypothesis that the apical organ of Hoplonemertea is reduced and the frontal organ appears de novo has neither been proposed by anyone nor confirmed by any observations, but this statement is accepted a priori without any evidence (perhaps, because reduction of the apical organ is observed in the larvae of other Spiralia). Our data do not support this hypothesis and allow us to put forward the hypothesis that the larval apical organ remains and is modified into a frontal organ in adults.

Our analysis of the hoplonemertean species *Q. stimpsoni* detected ultrastructural and immunoreactive similarities between the apical organ of the larva during the late stages of development (late rudiment and vermicular stages) and the frontal organ of the adult worm. Both organs feature an 8–12 cell apical plate that is associated with the excretory ducts of the frontal gland (in adult nemerteans this gland is commonly called ‘cephalic’). On the ultrastructural level, both apical and frontal organs are composed of columnar multiciliated cells with a single basal process that extends proximally from the cell body. In the apical organ, the excretory ducts of the gland pass only outside the apical plate. The excretory ducts of the frontal gland in adult hoplonemertean pass both outside the apical plate of the frontal organ and between the apical plate cells; the ducts open into different body parts along the entire gland (current study and [75]). The cell composition and ultrastructure of the larval frontal gland in the late rudiment stage larvae and the adult worms are similar.

Immunocytochemical studies detected positive signals for serotonin and acetylated tubulin in the apical plate and frontal organ cells featuring single axons. In both organs, only some axons form contacts with the subepithelial nerve plexus. In the adult worm, the frontal organ is not associated with any of these neurons (Fig. 8d), similar to that in the vermicular stage (Fig. 8c-c2). However, during the early and late rudiment stages, the apical plate is associated with the apical neurons (see the previous paragraph). Although the planktonic vermicular stage of the hoplonemerteans is considered the final larval stage before settlement, in *Q. stimpsoni* and other hoplonemerteans it clearly exhibits juvenile morphology [49, 76]. According to traditional views, such juveniles should not have a larval apical organ (at least, we do not know of any cases in other Spiralia), but our observations indicated an apical organ in the vermicular stage. The apical organ of this stage has features from early larval stages (apical neurons, paired subapical-plate neurons, and apical tuft) and the adult frontal organ (all apical plate cells are 5HT-lir, and their processes are involved in the formation of the subepithelial nerve plexus); thus, this organ can be considered as transitional between the apical and frontal organs.

These findings indicate that the frontal organ of adult hoplonemerteans is most likely the remodeled apical organ of the larvae, whereas the frontal gland is an obligatory component of both the apical and frontal organs. Interestingly, in *C. epialti* juveniles, the apical plate disappears [39], and *Carcinonemertes* is characterized by the absence of a frontal organ in the adult state, although the frontal gland is well-developed [77]. The apical organ of the larvae of another symbiotic nemertean, *Malacobdella grossa*, also appears to be reduced because the adult lacks a frontal organ [78]. Among the Hoplonemertea, the frontal organ and the frontal gland are absent in all pelagic forms and characterized by a reduction of the cephalic part of the body and the sensory organs that are typically positioned there [79].

**Fig. 11:**
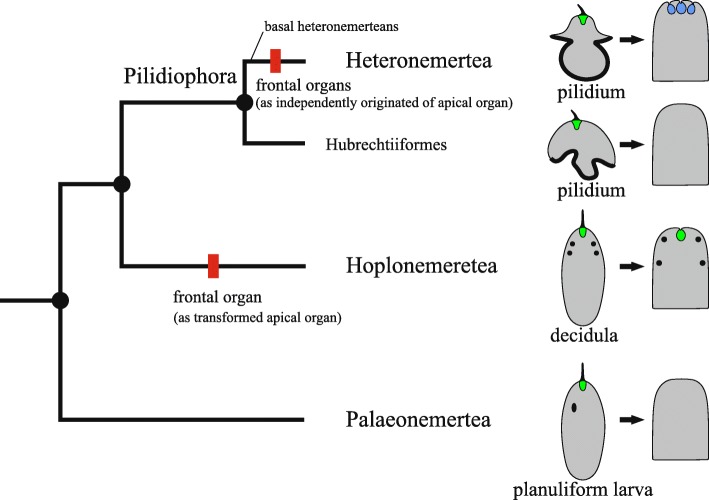
Distribution of larval types and frontal organs among major nemertean clades. Left: phylogenetic relationships among major nemertean clades (phylogeny modified after Andrade et al. [51] and Kvist et al. [52]). Red rectangles mark synapomorphies. Right: nemertean larvae (apical organs are marked with green) and heads of adult worms (hoplonemertean frontal organ is marked with green; heteronemertean frontal organ is marked with blue)

We are inclined to believe that the partial transformation of the larval apical organ into the frontal organ in adults is a hoplonemertean synapomorphy that does not occur outside this class. The larval apical organ of the palaeonemerteans completely disappears, and palaeonemerteans do not possess a frontal organ (Fig. 11), although the frontal gland may be developed [75, 79]. The heteronemertean frontal organs (typically three, rarely one or two) are not related to the apical organ in the pilidium larva. Riser [41] suggested that heteronemertean frontal organs originated independently of the hoplonemertean frontal organ (Fig. 11) and this is confirmed by the fact that the structure of these organs differs significantly between the heteronemerteans and the hoplonemerteans [80, 81]. Our analysis did not detect serotonin in three frontal organs of the heteronemertean *Kulikovia alborostrata* (Fig. 10b). In *Hubrechtella*, the basal group within the Pilidiophora (Fig. 11), the apical organ is present in the pilidium larvae, but the frontal organ is not developed in adult worms. In *Riserius pugetensis* and *Valencinura bahusiensis* frontal organs are also absent, and according to cladistics analysis [43], this state is primary (plesiomorphic) for these basal heteronemerteans. An alternative hypothesis for the homology of the frontal organ across nemerteans is less parsimonious, as it proposes independent reductions of this organ in Hubrechtiiformes, *Riserius*, and *Valencinura*. These data constitute additional evidence that the frontal organ of the heteronemerteans arose independently from the frontal organ of the hoplonemerteans.

## Conclusions

This study examined the development of the larval apical organ and the structure of the adult frontal organ in *Quasitetrastemma stimpsoni*. During the early stages of larval development, the apical plate cells are innervated by apical neurons, which is a common feature in spiralian larvae. During later larval development, serotonin appears in the apical plate cells, while the connection with apical neurons disappears. The apical plate cells of the adult worm also contain serotonin and form tiny processes that contribute to the formation of the subepithelial nerve plexus, suggesting that the adult frontal organ is a component of the peripheral nervous system. We propose that the common structural features of the apical organ in late swimming stages are preserved with small modifications in the frontal organ of adult worms. Specifically, both organs composed of apical plate cells surrounded by the ducts of frontal gland cells.

Our data appear to indicate that the hoplonemertean frontal organ is not formed de novo as in the heteronemerteans; instead, it is the transformed apical organ of the larva and free-swimming juvenile. It appears that this transformation is a unique feature of the hoplonemerteans and not found outside of this group. The complete reduction of the larval apical organ during metamorphosis and the absence of the frontal organ appears to represent the plesiomorphic state in the nemerteans, which is found in Palaeonemertea, the most basal group of Nemertea.

## Materials and methods

Mature *Q. stimpsoni* were collected in June and August of 2006–2009 in the Vostok Bay (Peter the Great Bay) of the Sea of Japan among rhizoids of the brown algae *Saccharina japonica* at a depth of 1 to 2 m. The larvae were obtained as described by Chernyshev [48]. In the current study, the terminology of Hiebert et al. [49] for nemertean decidula larval stages is used: (1) “early rudiment” stage, 36–48 hpf larvae; (2) “late rudiment” stage, 3–4 dpf larvae; and (3) “vermicular” stage, 7 dpf ‘larvae’. For comparative analysis, *pilidium gyrans* larvae (description see [82]) and heteronemertean *Kulikovia alborostrata* collected in the Vostok Bay were studied. To examine the frontal organ, the adult worms (approximately 3 cm in length) were anesthetized in 7% magnesium chloride and processed by dissecting the head.

For transmission electron microscopy (TEM) studies, the larvae collected at different post-fertilization times (36 hpf, 48 hpf, 3–4 dpf) and the heads of the adult animals were fixed in 2.5% glutaraldehyde in phosphate-buffered saline (1× PBS) (pH 7.4) and post-fixed with 1% osmium tetroxide in 1× PBS for 1 h. The fixed material was dehydrated in an ethyl alcohol and acetone series and embedded in Epon-Araldite resin (EMS). Transverse and longitudinal thin (60–70 nm) sections were made with an Ultracut E (Reichert) and stained with 1% uranyl acetate and 0.35% lead citrate solutions. The material was examined using the Libra 120 (Zeiss) transmission electron microscope (TEM).

For the confocal laser-scanning microscopy (CLSM) analysis, the larvae collected at different post-fertilization times (48 hpf, 70 hpf, 7 dpf) and the heads of the adult animals were fixed for 1–3 h at room temperature (RT; approximately 21 °C) with 4% paraformaldehyde in 1× PBS and then rinsed with 1× PBS. The samples were incubated in blocking buffer (BB; 10% normal donkey serum, 1% Triton X-100, 1% BSA in 1× PBS, and NaN_3_) overnight at RT. Primary antibodies (Abs) were dissolved in the BB, and the samples were incubated with primary Abs for 2 d (larval samples) or 5 d (adult animal samples). The following primary Abs were used: mouse anti-acetyl α-tubulin Abs (Sigma, 1:2000) and rabbit/goat polyclonal anti-5-HT Abs (Immunostar, 1:2000). The donkey 488-, 555-, and 647-conjugated Alexa secondary Abs (Invitrogen, 1:1000) were used for detection in combination with DAPI. Processed slices were mounted with glycerol-mounting medium (Merck). Images were acquired with a Zeiss LSM 710 confocal microscope. IMARIS (Karolinska University) and ImageJ software were used for image processing and analysis. The Bitplane IMARIS software was also used for 3D visualization and the analysis of confocal stacks. Images were further processed with Adobe Illustrator CS6 (Karolinska University) and Photoshop CS2 to adjust the contrast and brightness and to create digital line drawings.

## Data Availability

All data supporting the findings of this study are included in this published article.

## Abbreviations

5-HT-lir: 5-hydroxytryptamine-like immunoreactive
Abs: Primary antibodies
CLSM: Confocal laser-scanning microscopy
dpf: Days postfertilization
ECM: Extracellular matrix
hpf: Hours postfertilization
RT: Room temperature
TEM: Transmission electron microscopy
